# Septic Shock and Sepsis Syndrome in Obstetric Patients

**DOI:** 10.1155/S1064744994000645

**Published:** 1994

**Authors:** Peter G. Pryde, Bernard Gonik

**Affiliations:** Department of Obstetrics and Gynecology Division of Maternal and Fetal Medicine Wayne State University School of Medicine 4707 St. Antoine Boulevard Detroit MI 48201 USA

## Abstract

Septic shock is a life-threatening clinical syndrome that, despite its rare occurrence in obstetrics, remains a leading cause of maternal mortality. Its pathophysiology is explained by a profound systemic response to a complex variety of host cellular and humoral mediators elaborated after exposure to microbial toxins. Early recognition, prompt diagnostic workup, and immediate initiation of therapy improve outcomes. Therefore, recent publications have popularized the concept of the “sepsis syndrome,” a preshock list of clinical criteria associated with progressive sepsis. Needed diagnostic studies should never be withheld because of “pregnancy concerns.” With critically ill patients, the risk-to-benefit ratio supports the use of these diagnostic studies in almost all circumstances. Standard therapy is directed principally at restoring tissue perfusion by intravascular volume expansion and in some instances vasoactive pharmacological intervention. Simultaneously, identification of the source of infection and commencement of appropriate empiric antibiotic treatment are critical. In some cases, surgical abscess drainage or debridement of infected necrotic tissue will need to be considered. Novel approaches to treatment that attempt to reduce the systemic response to microbial toxins are promising and under active investigation. Pregnancy-specific considerations include the following: 1) initial signs or symptoms of septic shock may be masked by normal physiologic alterations of pregnancy; 2) a mixed polymicrobial group of organisms, consistent with lower genital tract flora, should be anticipated; and 3) initial therapy should be directed at maternal concerns since adverse fetal effects are most likely the result of maternal decompensation.


Infectious Diseases in Obstetrics and Gynecology 2:190–201 (1994)
© 1994 Wiley-Liss, Inc.

### Septic Shock and Sepsis Syndrome in Obstetric Patients

**Peter G. Pryde and Bernard Gonik**

*Department of Obstetrics and Gynecology, Division of Maternal and Fetal Medicine, Wayne State University School of Medicine, Detroit, MI*

Abstract

**Septic shock is a life-threatening clinical syndrome that, despite its rare occurrence in obstetrics, remains a leading cause of maternal mortality. Its pathophysiology is explained by a profound systemic response to a complex variety of host cellular and humoral mediators elaborated after exposure to microbial toxins. Early recognition, prompt diagnostic workup, and immediate initiation of therapy improve outcomes. Therefore, recent publications have popularized the concept of the "sepsis syndrome," a preshock list of clinical criteria associated with progressive sepsis. Needed diagnostic studies should never be withheld because of "pregnancy concerns." With critically ill patients, the risk-to-benefit ratio supports the use of these diagnostic studies in almost all circumstances. Standard therapy is directed principally at restoring tissue perfusion by intravascular volume expansion and in some instances vasoactive pharmacological intervention. Simultaneously, identification of the source of infection and commencement of appropriate empiric antibiotic treatment are critical. In some cases, surgical abscess drainage or debridement of infected necrotic tissue will need to be considered. Novel approaches to treatment that attempt to reduce the systemic response to microbial toxins are promising and under active investigation. Pregnancy-specific considerations include the following: 1) initial signs or symptoms of septic shock may be masked by normal physiologic alterations of pregnancy; 2) a mixed polymicrobial group of organisms, consistent with lower genital tract flora, should be anticipated; and 3) initial therapy should be directed at maternal concerns since adverse fetal effects are most likely the result of maternal decompensation.** © 1994 Wiley-Liss, Inc.

Key words

Pregnancy, diagnosis, therapy

The term *sepsis* refers to the presence of pyogenic or other pathogenic organisms and their toxins in tissues or in the blood. *Shock* is the inability, regardless of the underlying cause, of the circulatory system to maintain adequate cellular perfusion. Inadequate perfusion at the cellular level leads to membrane dysfunction, metabolic derangement, and finally cell death. The clinical definition of shock includes foremost hypotension [typically, a systolic blood pressure (BP) of <90 mmHg or a >40 mmHg decrement in baseline systolic BP] and can be accompanied by laboratory evidence of multiple organ injury.[1] If inadequately treated, the patient will progress relentlessly, and ultimately irreversibly, to multiple-organ-system failure and death. When the *shock syndrome* occurs as a result of sepsis, it is by definition *septic shock*.

Recently, in an attempt to identify patients at increased risk for developing septic shock, the *sepsis syndrome* was defined for a nonobstetrical population.[1] The diagnosis of this preshock syndrome is based on criteria consisting of readily recognized and easily acquired clinical data which include several of the more common manifestations of sepsis (Table 1). The objective of defining such a syndrome is to encourage earlier recognition of evolv-

Address correspondence/reprint requests to Dr. Bernard Gonik, Department of Obstetrics and Gynecology, Wayne State University School of Medicine, 4707 St. Antoine Boulevard, Detroit, MI 48201.

Received March 24, 1994

Accepted June 3, 1994

Review Article

**TABLE 1. Definition of the septic syndrome**

| |
|---|
| Clinical suspicion of infection |
| Fever or hypothermia |
| Tachypnea |
| Tachycardia |
| Manifestations of impaired organ-system perfusion |
| Central nervous system: altered mental status |
| Pulmonary shunt: hypoxemia |
| Anaerobic metabolism: elevated plasma lactate |
| Renal: oliguria |

Modified from Balk and Bone.[1]

ing sepsis with the goal of timely institution of therapeutic measures. This goal emphasizes the crucial concept that, in managing these potentially critically ill patients, early and aggressive treatment improves outcomes. Although not formally established for pregnancy, many of the above criteria would empirically apply to this patient population as well and should therefore be utilized.

### EPIDEMIOLOGY/RISK FACTORS

Evidence of localized infection is not uncommon on an obstetrical service. Rates of puerperal infections in indigent patients have been reported from 1 to 2% following vaginal delivery to a remarkable increase to 38.5–85% after cesarean section.[2,3] In antepartum patients, the most common infection is asymptomatic bacteriuria, with an incidence estimated at 4–7%, of which 40% of untreated cases are likely to develop pyelonephritis.[4–6] Other infections, which are considerably less common but significant in aggregate, include chorioamnionitis, septic abortion, wound infection, and pneumonia.

Despite the clearly elevated risk for developing localized infection during pregnancy or the puerperium, the life-threatening consequences are comparatively rare. Bacteremia occurs in only 8–10% of these infected patients.[7–9] More importantly, of those involving bacteremia, only 4–12% will develop clinical septic shock.[7–9] This is in contrast to a general medicine service on which only about 5% of patients have or develop signs of localized sepsis.[10] Yet, among these, nearly twice as many as in the obstetrical population become bacteremic (20%), and a much higher proportion of bacteremic patients (approximately 50%) manifest shock.[10] An additional important distinction between obstetrical and general medical patients is that the obstetrical patient is much less likely to die of complications of septic shock. Mortality rates with this condition have been estimated at approximately 3% compared with approximately 50% in obstetrical and nonobstetrical patients, respectively.[7–9,11] Nevertheless, given the relative rarity of maternal death in general, we should consider septic shock among its leading causes.[12]

### PATHOGENESIS OF SEPTIC SHOCK

Shock associated with sepsis can be caused by a variety of pathologic phenomena. This discussion shall focus principally on the pathophysiologic events leading to shock which can be attributed to an overwhelming systemic immune response to infection. Still, it needs to be recalled that other mechanisms can be responsible.[10] For example, hypovolemic shock can occur under conditions of sepsis due to massive fluid accumulation at the local site of infection, from gastrointestinal losses, or rarely, due to hemorrhage at a vessel with mycotic aneurysm formation and rupture. Other nonimmunogenic causes of shock during sepsis that require consideration are cardiogenic causes. These include valve dysfunction due to bacteremic seeding of the endocardium and primarily diminished myocardial performance which can be secondary to myocarditis or metastatic bacterial pericarditis.

The most common and clinically important mechanism of septic shock is related to the profound systemic response to immunologic processes triggered by proliferating infecting organisms or their byproducts. The sequence begins with a focus of infection such as an abscess, pyelonephritis, peritonitis, chorioamnionitis, or cellulitis. The offending microorganisms then either invade the bloodstream (bacteremia) or locally proliferate and release immunoreactive mediators into the bloodstream. These mediators consist principally of structural components of microorganisms such as endotoxin (lipopolysaccharide) in gram-negative bacteremia, exotoxin (peptidoglycan/techoic-acid complex) in gram-positive infections, and polysaccharide substances in yeast sepsis.[13–15]

The host response to these immunoreactive microbial byproducts is manifested through the activation of a complex and mutually interactive array of host mediator cells and humoral homeostatic systems.[16–19] Exposed endothelium releases vasoactive eicosanoids and nitric oxide as well as interleukins and procoagulant. Activated neutrophils produce and release platelet-activating factor, a variety of interleukins, vasoactive arachidonic-acid metabolites, lysosomal enzymes, and toxic molecular-oxygen derivatives. Activated macrophages principally release cachectin, also known as tumor necrosis factor, and histamine. Many of these host-cell products, in addition to potentiating one another's state of "activation," enhance microbial-mediated activation of several humoral-defense processes including the complement, coagulation, kinin, and adrenocorticotropic hormone (ACTH)/endorphin systems. The activated humoral systems in turn potentiate immune-cell activation.

While these host responses generally serve useful *local* antimicrobial functions, it is evident that with overwhelming infection they are activated *systemically* to an extent that their byproducts initiate and propagate the cascade of events that progress to the state of shock.[16,17] For example, local activation of the complement system enhances the bactericidal activity of circulating neutrophils. However, it also triggers intravascular events that, when elaborated at the systemic level, result in microvascular instability and histamine-mediated vasodilation.[10] Similarly, activated neutrophils produce numerous substances, listed above, that contribute to host defense by killing phagocytized microbes.[20] Yet, when systemically activated, these cells are observed to clump and adhere to vascular endothelium where their released byproducts are destructive to "bystander" host endothelium. The resulting microvascular damage contributes to several phenomena causing diffuse capillary leak. The products of activated leukocytes may also participate with immune-complex precipitants and humoral vasospastic substances in the complex events leading to specific end-organ damage such as acute respiratory disease syndrome (ARDS) and acute tubular necrosis.[21,22] The activated ACTH/endorphin system, which provides modulation of pain and discomfort to the host, may at high levels contribute to vasodilatation, diffuse capillary leak, and hypotension.[23] Finally, the coagulation system, which has an obvious homeostatic function when activated at the location of microbial-mediated tissue destruction, can be systemically activated resulting in thrombocytopenia from widespread platelet aggregation.[24] In many cases, frank disseminated intravascular coagulation is observed. Also activated in the coagulation cascade is the kinin system which elaborates the powerful vasodilator bradykinin. This substance potentiates the profoundly decreased systemic vascular-resistance (SVR) characteristic of septic shock.[11]

The cumulative impact of this overpowering immune response is a markedly decreased SVR and increased capillary permeability.[10,11,16] Together these cause decreased effective circulating blood volume and simultaneously diminished preload and afterload. The intact autonomic nervous system reflexly responds to this stimulus with an enormous output of catecholamines which stimulates a compensatory increase in cardiac output and regional increases in vascular resistance.[10,11,16]

Additional endogenous substances which appear to play an increasingly important role in later stages of septic shock are the myocardial depressant factors (MDF).[25] The chemical nature, biological source, and relevance of these substances to human septic shock remain to be fully characterized. However, because myocardial depressant activity seems to parallel serum levels of pancreatic lysosymes, these are considered possible candidates.[26] If so, MDF are presumably released under conditions of shock-related pancreatic ischemia.[10]

### CLINICAL FEATURES OF SEPTIC SHOCK

Septic shock can be divided conceptually into 3 clinical phases which together represent a stepwise deterioration of clinical status.[27] These phases are labeled 1) "early" (warm), 2) "late" (cold), and 3) "secondary" (irreversible) shock. In an effort to encourage earlier therapeutic intervention, which almost certainly improves outcomes, Balk and Bone[1] have recently defined the previously mentioned "preshock" phase which they have called "sepsis syndrome."

### Early Septic Shock

The earliest clinical sign of bacteremia is often extremes of body temperature. Typically, there is fever in excess of 40.6°C, although occasionally hypothermia is observed.[10] Tachycardia, warm extremities, and shaking chills will accompany these temperature changes. Symptoms may be nonspecific such as agitation, malaise, nausea, and diarrhea. However, a virtually uniform early symptom of which clinicians must be aware is dyspnea.[10]

Paradoxically, despite the predominant nature of this complaint, there are often minimal associated physical examination findings. This symptom is typically accompanied by tachypnea which needs to be regarded as a worrisome sign possibly presaging the onset of early ARDS. In some instances, there will be a rapid evolution of further clinical signs suggesting end-organ hypoperfusion, including mental status changes, and oliguria.[11] Concomitantly, or soon after these signs are recognized, the onset of hypotension is likely to be observed.[10,11]

This array of clinical findings represents the "warm" or vasodilated phase of septic shock. Hemodynamically, it is characterized by a profoundly hyperdynamic state with increased cardiac output and markedly diminished SVR.[10,11,16] When the reduction of SVR exceeds the physiologic capacity for compensatory increases in cardiac output, hypotension occurs. Laboratory findings may initially show a paradoxically decreased white blood cell (WBC) count due to the characteristic aggregation and endothelial adherence of activated neutrophils.[17] Platelets may also be decreased as an early manifestation of systemic activation of the coagulation cascade.[24] Electrolytes, transaminases, and renal function tests will initially be normal in most instances. In some cases, there may be transiently elevated serum glucose due to the high levels of circulating catecholamines and possibly decreased tissue utilization.[28] Arterial blood gasses in the early tachypneic phases will show only respiratory alkalosis reflecting a postulated direct effect of endotoxin or other mediators on the respiratory center.[29]

### Late Septic Shock

As the syndrome progresses to the "late" phase of shock, worsening hypotension is observed. The extremities become cool and cyanotic because of reflex peripheral vasoconstriction. Frank oliguria and deteriorating mental status reflect increasingly severe hypoperfusion of the renal parenchyma and cerebral cortex, respectively. Respiratory failure may ensue.

Hemodynamic evaluation during this phase may show transiently improved SVR due to peripheral vasoconstriction. However, the cardiac index begins to decrease (probably from a combination of vasoconstrictor-mediated increases in right and left ventricular afterload in addition to the escalating influence of MDF),[24,26] and consequently hypotension persists. Laboratory parameters at this point are more clearly abnormal. The leukopenia often manifested in earlier stages of septic shock is typically reversed, and a marked leukocytosis is observed. Thrombocytopenia may become more severe with additional derangements in coagulation parameters indicating evolving disseminated intravascular coagulation (DIC).[24] Serum electrolytes now show an anion gap with decreased bicarbonate and elevated blood lactate levels indicating metabolic acidosis from inadequate oxygen delivery and tissue extraction. Serum glucose may fall while transaminases rise, indicative of evolving hepatic dysfunction secondary to hypoperfusion ("shock liver").[30] Mixed venous oxygen saturation levels may be increased despite relatively high cardiac output suggesting a significant contribution of blood flow maldistribution and possibly a metabolic block to oxygen and other substrate utilization at the cellular level.[31] The arterial blood gas confirms metabolic acidosis and, in cases of developing ARDS, demonstrates hypoxia of variable severity.[16,21]

In some cases the condition continues to deteriorate with progression to severe ARDS and/or multiple-organ-system failure.[10,16] ARDS is a condition of noncardiogenic (or "capillary-leak") pulmonary edema. The diagnosis is made on the basis of progressive hypoxemia with characteristic diffuse pulmonary infiltrates on chest X-ray, decreased pulmonary compliance, and a normal pulmonary capillary wedge pressure (PCWP).[21] The term *multiple-organ-system failure* refers to the clinical detection of dysfunction in 2 or more organ systems, e.g., central nervous system, renal, respiratory, gastrointestinal, coagulation.[32] In patients dying of multiple organ failure, there is a characteristic sequence of pulmonary followed by hepatic and renal failure. Clinical outcomes are directly correlated with the number of organs failing. Reported mortality ranges from 50% with ARDS alone to 80–100% when 3 or more organs fail.[33]

### Secondary Septic Shock

Myocardial depression becomes a prominent feature of prolonged "late" phase shock as the patient slips into "secondary" shock.[11,16,25,26] Widespread terminal vasodilatation and pooling of the effective circulatory volume also occur. The hemodynamic

**TABLE 2. Hemodynamic, ventilatory, and hematologic parameters in pregnancy**

| Parameter | Nonpregnant | Pregnant |
|---|---|---|
| Central venous pressure | 1–10 mmHg | Unchanged |
| PCWP | 3–10 mmHg | Unchanged |
| Cardiac output | 4–7 l/min | Increased 30–45% |
| SVR | 770–1,500 dyne/sec/cm$^{-5}$ | Decreased 25% |
| Pulmonary vascular resistance | 20–120 dyne/sec/cm$^{-5}$ | Decreased 35% |
| Arterial $pO_2$ | 90–95 mmHg | 104–108 mmHg |
| Arterial $pCO_2$ | 38–40 mmHg | 27–32 mmHg |
| Arterial pH | 7.35–7.40 | 7.40–7.45 |
| A/V oxygen difference | 4–5.5 ml/dl | Slightly decreased or unchanged |
| Oxygen consumption | 173–311 ml/min | 249–331 ml/min |
| Creatinine clearance | 80–110 ml/min | Increased 35–50% |
| Hemoglobin | 13.5 g/dl | 12 g/dl |
| Platelets | 270–280 (× $10^9$/l) | 250–265 (× $10^9$/l) |
| Fibrinogen | 170–410 mg/l | 264–615 mg/l |

picture shows remarkable decrements in cardiac output in the face of profoundly low SVR and extreme hypotension. Prolonged diffuse tissue hypoxia is reflected clinically by obtundation and anuria. Spontaneous bleeding may occur. Laboratory findings show severe metabolic acidosis, coagulopathy, and renal failure. In cases of lethal ARDS, worsening hypoxia with respiratory acidosis superimposed on metabolic acidosis is observed despite maximum ventilatory support.[21] By this phase, shock is usually irreversible and death inevitable.[32,33]

### ASPECTS OF SEPTIC SHOCK SPECIFIC TO PREGNANCY

As mentioned, the reported mortality rates in pregnant and puerperal women with septic shock are considerably lower than in their counterparts on general medical or surgical services. Several explanations for this have been suggested. Foremost, obstetrical patients on the whole are younger and healthier than hospitalized medical patients. As such, they rarely have underlying debilitating diseases that are known to complicate sepsis and increase mortality. Furthermore, in obstetrical infections, the pathogens tend to be "garden-variety" endogenous flora that are less often resistant to standard antibiotic regimens. Finally, these infections are usually etiologically straightforward and amenable to medical or, if necessary, surgical intervention.

Because of 1) the well-described physiologic adaptations of virtually every organ system to normal pregnancy and 2) the less well-defined pregnancy-related immunologic modifications, differences in response to septic shock in gravid women compared with age-matched healthy nongravid women would be anticipated. However, probably due to the infrequent occurrence of septic shock in pregnancy, this area has received little attention in the literature. Nevertheless, data are available regarding respiratory, hemodynamic, and other physiologic alterations in normal human pregnancy (Table 2).[34,35] It is important that caretakers of critically ill pregnant women be cognizant of such data and apply them for guidance of therapeutic interventions.

Using experimental animals, investigators have studied septic shock in pregnant animals compared with nonpregnant controls. The preponderance of data indicates an increased susceptibility to the pathologic effects of endotoxin in several mammalian species at term.[36–38] Although the pathophysiologic basis for such observations remains speculative, it has been suggested on the basis of these results that gravidas be considered compromised hosts. Regarding the effect of endotoxin on the fetus, data in experimental animals are less clear. Sheep fetuses were shown in one study to tolerate endotoxin in doses 10-fold higher than those that would be lethal to their mothers.[38] The investigators hypothesized that the immature status of the fetus modified its vasoactive response, protecting it from lethal hypotension. In a separate study, the fetus of a pregnant baboon showed signs of rapid deterioration and asphyxia following maternal administration of endotoxin.[37] In this study, the fetal effects were attributed to maternal endotoxin-mediated hypotension and uterine hypercontractility, both of which contribute to hypoperfusion of the uteroplacental unit.

### MANAGEMENT OF SEPTIC SHOCK

The principles of septic shock management in the pregnant or puerperal woman are similar to those in any septic patient.[27] The goal is to maximize tissue oxygen delivery.[10,11,16,27] This is accomplished by establishing and maintaining optimal functional levels of 1) circulating fluid volume, 2) cardiac output, 3) blood oxygen carrying capacity, and 4) pulmonary gas exchange. Also of critical importance is to identify the potential sources of sepsis and perform appropriate diagnostic evaluations. Finally, empiric antibiotic therapy is initiated targeting the clinically most likely pathogens. If a surgically significant source is apparent, drainage should be accomplished.

During pregnancy, many difficult management issues are amplified by concerns about the fetus. Still, optimal therapy for the mother remains the 1st priority. Fetal compromise under these circumstances is directly attributable to maternal cardiovascular decompensation and the attendant decrease in uteroplacental blood flow. Hence, improvements in maternal hemodynamic stability will have positive effects on the fetus. In addition, efforts to intervene on behalf of the fetus by operative delivery of an unstable mother may end in catastrophic results for both the fetal and the maternal patients. The obvious exception to this axiom is when the source of sepsis is the fetal compartment in which case delivery becomes a critical part of therapy, i.e., surgical "drainage."

The traditional therapeutic interventions employed in septic shock management are aimed at reversing the physiologic aberrations encountered in septic shock. Decrements in effective circulating volume and oxygen delivery to tissues are treated by fluid replacement, pressor and inotropic drug therapy, and blood transfusions. Coagulopathy is managed with selective transfusions. Support for pulmonary dysfunction is initiated by increased fractional inspiratory oxygen concentration and, if necessary, mechanical ventilation. With recent insights regarding the pathophysiologic mechanisms of hemodynamic deterioration in septic shock, several innovative avenues of therapy have been suggested and investigational protocols initiated based on attempts to arrest or modify the overzealous activation of host immune cells and humoral systems. Such approaches include novel antiendotoxin, antieicosanoid, and antiendorphin pharmacotherapies.

### Circulatory Volume Expansion and Hemodynamic Monitoring

The initial resuscitation for septic shock is by aggressive circulatory volume expansion. Such therapy is essential in all cases, and the sooner it is initiated the better. In responding patients, clinical improvement is indicated by increments in cardiac output and tissue oxygen consumption, with a concomitant decrease in arterial lactate levels. Remarkable quantities of replacement fluid are often needed due to the enormous redistribution of circulatory blood volume by the combined effect of extensive vasodilatation and the "third-space" loss of previously intravascular fluids attributed to increased capillary permeability.

Most authors[27,39,40] agree that the initial fluid bolus should be crystalloid (0.9% saline or lactated ringers) at a volume of 1–2 l. While the bolus is being infused, it is recommended that central hemodynamic monitoring be initiated. For this, a *flow-directed pulmonary-artery catheter* is recommended which allows the most accurate available assessment of left ventricular filling pressures (or preload) to guide fluid management.[41] A variety of protocols have been described for monitoring fluid challenges after pulmonary-artery catheterization. The popular method of Shubin and associates[42] includes serial boluses of 200 cc of intravenous (IV) fluid over 10 min. Measurements of PCWP are determined between boluses. For pressure increases <7 mmHg, boluses are repeated. The aim of therapy is a PCWP of approximately 10–15 mmHg which, in septic shock patients, represents the point at which left ventricular preload is optimized according to the Frank-Starling law.[43] Additional information provided by the pulmonary-artery catheter includes estimation of the cardiac output, measurement of mixed venous oxygen saturation, and data for calculations of vascular resistance and oxygen utilization. All of these parameters provide valuable information not only for fluid management but for the guidance of all hemodynamic interventions applied to shock patients.

### Vasoactive Pharmacotherapy

In patients who fail to respond adequately to optimization of preload with pulmonary-artery-catheter-guided fluid management, the next step in septic shock therapy is the use of exogenous catecholamines or sympathomimetic agents. Most clinicians employ dopamine as the first-line vasoactive agent.[16,27,39,40,44] A feature unique to this drug is its dose-related catecholamine-receptor selectivity. At low infusion rates (0.5–3.0 μg/kg/min), the observed physiologic actions are primarily dopaminergic receptor mediated and are characterized by renal, splanchnic, and cerebral vasodilatation.[45] The attendantly increased renal perfusion at such doses leads to improved urinary output. At higher infusion rates (5–10 μg/kg/min), β-adrenergic-mediated effects predominate. Inotropic cardiac effects are observed without significant peripheral vasoconstriction. However, as rates of infusion approach and exceed 15 μg/kg/min, α-receptor-mediated effects become dominant. Under these conditions, there is widespread peripheral vasoconstriction and reversal of the selective vasodilatory effects of lower-dose dopamine.[46] The physiologic results at high doses, all of which are countertherapeutic in septic shock patients, are diminished systemic perfusion as well as increased afterload and cardiac work.[44]

Other sympathomimetic drugs that may be used in the setting of septic shock include dobutamine and norepinephrine. Dobutamine, due to its predominance of β-1 and β-2 adrenergic effects combined with a relative absence of α-adrenergic effect, simultaneously provides inotropic support and decreased SVR.[44] This allows improved cardiac output without considerable increases in cardiac work or cardiac oxygen demand.[47] Hence, it may be an appropriate choice when low cardiac output becomes a dominant feature in patients with advanced septic shock. In contrast, norepinephrine is principally an α-adrenergic stimulatory catecholamine.[44] Its effects are peripheral vasoconstriction. In early septic shock, one of the pathophysiologic phenomena with which the physician must contend is profound systemic vasodilation. Some investigators[44,48] have suggested that norepinephrine in combination with low-dose dopamine (to preserve renal blood flow) be used to ameliorate this problem. An animal model has documented an improvement of cardiovascular parameters while improving renal blood flow on such a regimen, but a therapeutic benefit in humans remains to be demonstrated.[49]

### Therapy for Respiratory Failure

As mentioned, respiratory failure in the form of ARDS is a common complication of septic shock.[21] The diagnosis is usually made on the basis of progressive hypoxemia, diffuse infiltrates on the chest X-ray, and decreased pulmonary compliance despite a normal PCWP. It should be kept in mind that ARDS is not a disease entity but a unifying pulmonary complication of multiple varied critical disease processes. Therefore, therapy is *supportive* with the goal of maintaining ventilation and oxygenation. While the primary disease (the septic source) is treated and reverses, the secondary pulmonary injury will generally resolve. As with all types of respiratory failure unresponsive to noninvasive therapy, treatment is initiated with intubation followed by ventilation using high-fractional inspiratory concentrations of oxygen. Subsequently, an attempt is made to rapidly decrease inspired oxygen levels to nontoxic doses (generally thought to be <50%). An arterial sampling catheter is usually placed for serial blood gas monitoring to guide ventilator changes. Frequently, in cases of ARDS, achieving nontoxic inspired oxygen levels requires the employment of positive-end expiratory pressure (PEEP).[16,21] The limiting factors with PEEP are related to barotrauma (including pneumothorax and alveolar overexpansion) and negative influences on cardiac output, both of which occur at higher PEEP pressures.[16,21]

### Identification of Septic Source and Antibiotic Initiation

Optimal therapy for the septic shock patient requires rapid and aggressive initiation of treatment directed at the underlying septic source. Thus, simultaneously with efforts to support cardiovascular and respiratory status of the patient, a careful investigation into the location and cause of the responsible infection needs to be performed. As quickly as possible, *empiric* antimicrobial therapy must then be started.

The diagnostic workup for a septic source is of crucial importance as its findings 1) guide antibiotic choice and 2) may uncover a locus requiring surgical intervention for definitive therapy. In obstetrics, the source is frequently but not always obvious. In every case, a thorough evaluation is essential beginning with a complete physical examination and proceeding with directed laboratory evaluation, culture of appropriate body fluids, and in most instances performance of selected imaging techniques. In virtually all cases, the minimal evaluation should include a chest X-ray and microbiologic evaluation of the blood, urine, sputum, and any wound (including the postpartum endometrium) which may be present. When the patient is still pregnant, an additional etiology that requires strong consideration is amnionitis. This is easily evaluated by amniocentesis which, in the absence of definitive evidence of an alternative source, must be performed. Some cases may require ultrasound, computed tomography, or magnetic resonance imaging of the abdomen and pelvis when the specific source remains elusive or the physical examination strongly suggests an abscess in one of these cavities.

After completion of the sepsis workup, empiric antibiotic therapy is initiated without delay. Based on the likely sources of infection in the obstetrical patient, antimicrobial therapy must target a broad spectrum of bacteria including aerobic and anaerobic (gram-positive and gram-negative) bacteria. Most authors agree that combination parenteral therapy is most appropriate.[27,39,40] Although a variety of protocols have been suggested, a well-established regimen includes a penicillin at high dose, an aminoglycoside at the appropriate weight-adjusted dose, and an antianaerobic agent such as clindamycin or metronidazole. Because of the nephrotoxic risk of aminoglycoside therapy, it is essential that peak and trough levels be monitored and dosages adjusted accordingly with target levels of 6–10 and $<2$ μg/ml, respectively. In patients with preexisting or rapidly evolving renal insufficiency, an alternative antibiotic regimen substituting aztreonam for the aminoglycosides may be considered. This preserves gram-negative coverage while decreasing nephrotoxic risk. Empiric combination therapy is continued pending culture and sensitivity results. When available, these results allow adjustment of the regimen in order to be more antibiotic selective and specific-microbe directed.

### Surgical Intervention

A classical surgical principle maintains that "the treatment of abscess is incision and drainage." Recently, this dogma has been challenged under some circumstances such as an uncomplicated tubo-ovarian complex in which resolution with antibiotics alone has been well documented in many cases.[50] Still, when faced with septic shock with an identified source, most clinicians agree that drainage of infected fluid collections and removal of devitalized tissues should improve survival. Similarly, in patients in whom the septic source is not obvious, a delay in clinical improvement or progression to multiple-organ-system failure despite maximal medical therapy should suggest an undrained locus of infection.

In obstetrical patients with septic shock, there are several scenarios in which the physician must contemplate surgical intervention. Septic abortion, for example, requires prompt initiation of broad-spectrum antibiotics and hemodynamic stabilization. Subsequently, the uterus must be evacuated.[51] This should be done expeditiously and can be effected either by dilation and evacuation or by medical induction of labor. The former has the advantage of predictable and timely removal of the septic source. However, it requires operator experience which, for advancing gestational ages, is increasingly rare among obstetricians.

In patients presenting with chorioamnionitis, the obstetrician is faced with the difficult judgment of balancing maternal and fetal risks. After initial resuscitation of the mother, a plan must be made and initiated as to the route of delivery. When a vaginal delivery is imminent and both the maternal and fetal patients are stable, antibiotics should be initiated and a cesarean section often can be avoided. However, in cases remote from potential vaginal delivery, the risk of cesarean section to a critically ill parturient may be warranted. Our anecdotal experiences suggest that in such cases the improved chances for the fetus by effecting urgent delivery, coupled with the uncertain risk to the mother of delaying removal of the nidus of infection, justify cesarean section. A decision in some cases will be difficult and must be individualized. It is important to emphasize that delivery is usually *not* indicated for septic shock patients in whom the septic source is extrauterine, i.e., appendicitis, pyelonephritis, pneumonia.

A final scenario deserving mention is the postoperative cesarean section patient with clinical deterioration despite optimal antibiotic coverage. In some cases, the explanation is extensive myometrial microabscess formation and definitive therapy requires hysterectomy. Other conditions to be considered under such circumstances include septic pelvic-vein thrombosis (which in most cases responds when anticoagulation therapy is added to the antibiotic regimen) and episiotomy or other wound infections (which require subcutaneous and in many cases extensive fascial debridement).[52]

### Miscellaneous Therapeutic Concerns

Additional complications not unique to but frequently encountered in septic shock patients are acute renal failure and coagulopathy.[24,53] In all shock patients, an indwelling urinary catheter is placed for hourly assessment of urine production. This provides a clinical parameter for end-organ perfusion. As output begins to diminish serial serum creatinine, an examination of the urine sediment and comparison of urinary to plasma sodium and osmolality provide useful data for distinguishing prerenal from renal parenchymal damage (acute tubular necrosis) due to prolonged hypoperfusion. Patients with prerenal oliguria will have low fractional excretion of sodium with high urinary osmolality and will respond to intravascular volume expansion. Patients having acute tubular necrosis will have high fractional excretion of sodium, low urinary osmolality, and often "dirty" tubular casts in a spun urine. These patients typically remain oliguric but may manifest a nonoliguric renal insufficiency with progressive increments in serum creatinine. In most cases, the renal parenchymal damage is reversible with the resolution of the compromised hemodynamic state of shock, although the recovery may be sufficiently delayed to require short-term dialysis. Rarely, renocortical necrosis may occur, in which case the renal failure is most likely permanent.[53]

Coagulation abnormalities reflecting variable-severity DIC are a frequent finding in septic shock patients, and serial evaluation of platelets, fibrinogen, and fibrin degradation products is suggested.[24] In the absence of frank clinical bleeding, the management is mainly a reversal of the underlying condition after which the coagulopathy spontaneously resolves. However, when spontaneous bleeding does occur, e.g., bleeding mucous membranes, oozing at venipuncture or arterial-puncture sites, postoperative incisional bleeding, or when platelets fall below 20,000/ml (the level below which spontaneous internal hemorrhage is thought to occur), selective blood-product transfusion including an appropriate combination of platelets, fresh-frozen plasma, and cryoprecipitate may be indicated.[16]

### Controversial and Investigational Treatment Modalities

A longstanding controversy in the treatment of septic shock is the use of pulse-dose corticosteroids. The rationale for such an approach is firmly supported by the present conceptualization of overzealous host inflammatory-mediated pathophysiologic events culminating in clinical septic shock. Early experimental and clinical studies[54] reported increased survival with pharmacologic doses of steroids. However, more recent reports, including 2 large multicenter studies,[55,56] failed to demonstrate improved outcomes in steroid-treated septic shock patients. In fact, the subgroup of patients having renal insufficiency had apparently increased mortality in the steroid-treated group.[56] Similarly, in high-risk patients, no decrease in the progression to septic shock or the development of ARDS could be established with prophylactic high-dose steroids.[56] Consequently, few intensivists are recommending steroids for the treatment of septic shock except in patients with suspected adrenal insufficiency or other specific indications.

Endorphins are endogenous opiates that are known to be elaborated during sepsis.[23] They have potent vasodilatory effects and have been implicated as contributors to the hemodynamic instability characteristic of septic shock. Naloxone is an opiate antagonist that will reverse endorphin-induced hypotension in experimental animals.[23] However, both experimental and clinical studies have yielded inconclusive data regarding the hemodynamic benefits of naloxone administered for septic shock.[23,57] More work needs to be done prior to clinical implementation of this therapy. An additional concern about naloxone specific to pregnancy is the unexplained observation of gradual increases in steady-state endorphin levels with advancing gestation.[58] The importance of this phenomenon to normal physiology and its relevance under conditions of septic shock in pregnant women are not known.

Immunologic therapy for the treatment and prevention of septic shock is an area of active and promising investigation. Most attention in this area has been focused on antibodies directed against endotoxin.[59] Endotoxin, which is released during bacterial lysis in gram-negative septicemia, has been shown in experimental animals to produce many of the acute physiologic and long-term pathologic events observed in human septic shock.[59,60] Bioengineered antiendotoxin antibodies have been shown in these models to prevent some of these changes. The most efficacious antibodies seem to be those directed specifically toward the innermost portion (lipid A) of the complex endotoxin molecule. In contrast to the earlier constructed antibodies directed at the outermost species-specific oligosaccharide (O) region of the endotoxin molecule, the antilipid A antibodies bind to a wide spectrum of gram-negative organisms.[61] In clinical studies, such antibodies have demonstrated clear enhancement of survival and earlier reversal of organ dysfunction compared with placebo in series of medical and surgical patients with septic shock.[62,63] A reduction in incidence of septic shock and deaths related to sepsis was also demonstrated in high-risk surgical patients randomized to a regimen of prophylactic antiendotoxin.[62] Studies specifically in obstetrical patients are limited. One small mixed series[64] of obstetrical and gynecologic patients showed improved survival (93% vs. 54%) and shorter hospital stays in those administered an antilipopolysaccharide compared with those given no therapy. Other avenues of immune therapy under current investigation involve antibodies directed against macrophage-derived tumor necrosis factor[18] and efforts to derive nontoxic derivatives of lipid A to attenuate the systemic response to endotoxin.[65]

A final strategy for septic shock therapy by modification of the host response to septicemia is via the eicosanoid system. As mentioned, several eicosanoids (including a variety of prostaglandins, leukotrienes, and thromboxane) appear to be important mediators of septic shock.[10,11,16,17,20] Several studies[66,67] suggest that prostaglandin synthetase, leukotriene, and thromboxane inhibitors attenuate the hemodynamic response and improve survival in experimental endotoxic and bacteremic shock. Rigorous clinical trials of this application for these drugs are forthcoming, but initial data are promising.

### REFERENCES

1. Balk RA, Bone RC: The septic syndrome: Definition and clinical implications. Crit Care Clin 5:1–9, 1989.
2. Gibbs RS, Jones PM, Wilder CJ: Antibiotic therapy of endometritis following cesarean section. Obstet Gynecol 52:31–35, 1978.
3. Duff P: Pathophysiology and management of postcesarean endometritis. Obstet Gynecol 67:269, 1986.
4. Whaley P: Bacteriuria of pregnancy. Am J Obstet Gynecol 97:723–738, 1967.
5. Monson OT, Armstrong D, Pion RJ, et al.: Bacteriuria during pregnancy. Am J Obstet Gynecol 85:511–518, 1963.
6. Kass EH: Bacteriurea and pyelonephritis of pregnancy. Arch Intern Med 205:194–198, 1960.
7. Blanco JD, Gibbs RS, Castaneda YS: Bacteremia in obstetrics: Clinical course. Obstet Gynecol 58:621–625, 1981.
8. Ledger WJ, Norman M, Gee C, Lewis W: Bacteremia on an obstetric-gynecologic service. Am J Obstet Gynecol 121:205–212, 1975.
9. Bryan CS, Reynolds KL, Moore EE: Bacteremia in obstetrics and gynecology. Obstet Gynecol 64:155, 1984.
10. Sheagren JN: Shock syndromes related to sepsis. In Weingaarden JB, Smith LH Jr (eds): Cecil Textbook of Medicine. 18th ed. Philadelphia: W.B. Saunders, pp 1538–1541, 1988.
11. Parillo JE: Septic shock in humans: Advances in the understanding of pathogenesis, cardiovascular dysfunction, and therapy. Ann Intern Med 113:227–242.
12. Atrash HK, Koonin LM, Lawson HW, Franks AL, Smith JC: Maternal mortality in the United States, 1979–1986. Obstet Gynecol 76:1055–1060, 1990.
13. Morrison D, Ryan JL: Endotoxins and disease mechanisms. Annu Rev Med 38:417–432, 1987.
14. Wiles J, Cerra F, Siegel J, Broder J: The systemic response: Does the organism matter? Crit Care Med 8:55–60, 1980.
15. Sheagren J: *Staphylococcus aureus.* N Engl J Med 310:1368–1373, 1984.
16. Rackow EC, Astiz ME: Pathophysiology and treatment of septic shock. JAMA 266:548–554, 1991.
17. Jacobs R, Trubor D: Immune cellular interactions during sepsis and septic injury. Crit Care Clin 5:9–26, 1989.
18. Beulter B, Cerami A: Cachectin: More than a tumor necrosis factor. N Engl J Med 316:379–385, 1987.
19. Kilbourn RG, Belloni: Endothelial cell production of nitrogen oxides in response to interferon-gamma in combination with TNF, interleukin-1 or endotoxin. J Natl Cancer Inst 82:772–776, 1990.
20. Weissmann G, Smolen JE, Korchak HM: Release of inflammatory mediators from stimulated neutrophils. N Engl J Med 303:27–34, 1980.
21. Fein A, Lippman M, Holtzman H, Eliraz H, Goldberg S: The risk factors, incidence and prognosis of ARDS following septicemia. Chest 83:40–42, 1983.
22. Beaufils M, Morel-Maroger L, Sraer J, Kanfer H, Koürilsky O, Richet G: Acute renal failure of glomerular origin during visceral abscesses. N Engl J Med 295:185–189, 1976.
23. Holaday JW, Faden AI: Naloxone reversal of endotoxin hypotension suggests a role of endogenous endorphins in shock. Nature 275:450–451, 1979.
24. Oppenheimer L, Hryniuk W, Bishop A: Thrombocytopenia in severe bacterial infections. J Surg Res 20:211–214, 1976.
25. Parrillo JE, Burch C, Shelhamer JH, Parker MM, Natenson C, Shuette W: A circulating myocardial depressant substance in humans with septic shock. J Clin Invest 76:1539–1553, 1985.
26. Cunnion RE, Parillo JE: Myocardial dysfunction in sepsis. Crit Care Clin 5:99, 1989.
27. Gonik B: Septic shock in obstetrics. Clin Perinatol 13: 741, 1986.
28. Bessey P, Walters J, Aoki T, Wilmore E: Combined hormonal infusion stimulates the metabolic response to injury. Ann Surg 200:264–281, 1984.
29. Blair E: Hypocapnia and gram-negative bacteremia shock. Am J Surg 19:433–439, 1970.
30. Rawson J, Achord J: Shock liver. South Med J 78:1421–1425, 1985.
31. Astitz ME, Rackow EC, Falk JL, Weil MH: Oxygen delivery and consumption in patients with hyperdynamic septic shock. Crit Care Med 15:26–28, 1987.
32. Carrico CJ, Meakins JL, Marshall JC, et al.: Multiple-organ-failure syndrome. Arch Surg 121:196, 1986.
33. Knaus W, Draper E, Wagner D, Zimmerman J: Prognosis in acute organ-system failure. Ann Surg 202:685–693, 1985.
34. Clark SL, Cotton DB, Lee W, et al.: Central hemodynamic assessment of normal third trimester pregnancy. Am J Obstet Gynecol 161:1439, 1989.
35. Ramsay M: Appendix of normal values. In James DK, Steer PG, Weiner CP, Gonik B (eds): High Risk Pregnancy; Management Options. London: W.B. Saunders, pp 1259–1289, 1994.
36. Beller FK, Schmidt EH, Holzgreve W, et al.: Septicemia during pregnancy: A study in different species of experimental animals. Am J Obstet Gynecol 151:967, 1985.
37. Morishima HO, Niemann WH, James LS: Effects of endotoxin on the pregnant baboon and fetus. Am J Obstet Gynecol 131:899, 1978.
38. Bech-Jansen P, Brinkman CR, Johnson GH, et al.: Circulatory shock in pregnant sheep. Am J Obstet Gynecol 112:1084, 1972.
39. Lee W, Clark SL, Cotton DB, et al.: Septic shock during pregnancy. Am J Obstet Gynecol 159:410–416, 1988.
40. Pearlman M, Faro S: Obstetric septic shock: A pathophysiologic basis for management. Clin Obstet Gynecol 33:482–492, 1990.
41. Amin DK, Shah PK, Swan HJC: The Swan-Ganz catheter. J Crit Illness 1:24–69, 1986.
42. Shubin H, Weil MH, Carlson RW: Bacterial shock. Am Heart J 94:112, 1977.
43. Packman M, Rackow EC: Optimum left heart filling pressures during fluid resuscitation of patients with hypovolemic and septic shock. Crit Care Med 11:165–169, 1983.
44. Boyd JL, Stanford GC, Chernow B: The pharmacotherapy of septic shock. Crit Care Clin 5:133–150, 1989.
45. Goldberg LI: Dopamine: Clinical uses of an endogenous catecholamine. N Engl J Med 291:707–710, 1974.
46. Higgins TL, Chernow B: Pharmacotherapy of circulatory shock. Dis Mon 33:311–361, 1987.
47. Sonnenblick EH, Frishman WH, LeJemtel TH: Dobutamine: A new synthetic cardioactive sympathetic amine. N Engl J Med 300:17–22, 1979.
48. Desjars P, Pinaud M, Potel G, et al.: A reappraisal of norepinephrine therapy in human septic shock. Crit Care Med 15:134–137, 1987.
49. Schaler GL, Fink MP, Parrillo JE: Norepinephrine alone versus norepinephrine plus low-dose dopamine: Enhanced renal blood flow with combination pressor therapy. Crit Care Med 13:492–496, 1985.
50. Reed SD, Landers DV, Sweet RL: Antibiotic treatment of tubo-ovarian abscess. Am J Obstet Gynecol 164:1556, 1991.
51. Pritchard JA, Whalley PJ: Abortion complicated by *Clostridia perfringens* infection. Am J Obstet Gynecol 111: 484, 1971.
52. Duff P, Gibbs RS: Pelvic vein thrombophlebitis: Diagnostic dilemma and therapeutic challenge. Obstet Gynecol Surv 38:365, 1983.
53. Menashe P, Ross S, Gottlieb J: Acquired renal insufficiency in critically ill patients. Crit Care Med 16:1106–1109, 1988.
54. Shumer W: Steroids in the treatment of septic shock. Ann Surg 184:333–341, 1976.
55. Veterans Administration Systemic Sepsis Cooperative Study Group: The effect of high dose glucocorticoid therapy on mortality in patients with clinical signs of systemic sepsis. N Engl J Med 317:659–665, 1987.
56. Bone R, Fischer C, Clemmer T, Slotman G, Metz C, Balk R: Methylprednisolone in severe sepsis study group: A controlled clinical trial of high dose methylprednisolone in the treatment of severe septic shock. N Engl J Med 317:653–658, 1987.
57. Rock P, Silverman H, Plump D, et al: Efficacy and safety of naloxone in septic shock. Crit Care Med 13:28–33, 1988.
58. Genazzani AR, Facchinetti F, Parrini D: β-Lipotrophin and β-endorphin plasma levels during pregnancy. Clin Endocrinol 14:409, 1981.

59. Jacobson RL, Gonik B: Antiendotoxin: What role in ob-gyn? Contemp Obstet Gynecol 37:12–16, 1992.
60. Feely TW, Minty BD, Schudder CM, et al.: Studies of an antiendotoxin antibody in preventing the physiologic changes of endotoxemia in awake sheep. Am Rev Respir Dis 142:775, 1990.
61. McCabe WR, Kreger B, Johns M: Type specific and cross reactive antibodies in gram-negative bacteremia. N Engl J Med 287:261–267, 1972.
62. Baumgartner J, McCutchan J, Melle G, et al.: Prevention of gram-negative shock and death in surgical patients by antibody of endotoxin core glycolipid. Lancet 2:54–63, 1985.
63. Ziegler EJ, Fisher CJ Jr, Sprung CL, et al.: Treatment of gram-negative bacteremia and septic shock with HA-IA human monoclonal antibody against endotoxin. N Engl J Med 324:429–436, 1991.
64. Lachman E, Pitsoe SB, Gaffin SI: Antilipopolysaccharide immunotherapy in management of septic shock of obstetric and gynecologic origin. Lancet 1:981, 1984.
65. Takayama K, Qureshi N, Ribi E, Cantrell J: Separation and characterization of toxic and nontoxic forms of lipid A. Rev Infect Dis 6:439–443, 1984.
66. Cefalo RC, Lewis PE, O'Brian WF, et al.: The role of prostaglandins in endotoxemia: Comparisons in response in the nonpregnant, maternal, and fetal models. Am J Obstet Gynecol 137:53, 1980.
67. Haupt M, Jastremski M, Clemmer T, Metz C, Brown B: Effects of ibuprofen in patients with severe sepsis syndrome. Chest 96:291, 1989.

## References

[PDF_01029] Astiz M. E., Rackow E. C., Falk J. L., Kaufman B. S., Weil M. H. (1987). Oxygen delivery and consumption in patients with hyperdynamic septic shock.. Crit Care Med.

[PDF_00976] Atrash H. K., Koonin L. M., Lawson H. W., Franks A. L., Smith J. C. (1990). Maternal mortality in the United States, 1979-1986.. Obstet Gynecol.

[PDF_01124] Baumgartner J. D., Glauser M. P., McCutchan J. A., Ziegler E. J., van Melle G., Klauber M. R., Vogt M., Muehlen E., Luethy R., Chiolero R. (1985). Prevention of gram-negative shock and death in surgical patients by antibody to endotoxin core glycolipid.. Lancet.

[PDF_01004] Beaufils M., Morel-Maroger L., Sraer J. D., Kanfer A., Kourilsky O., Richet G. (1976). Acute renal failure of glomerular origin during visceral abscesses.. N Engl J Med.

[PDF_01051] Bech-Jansen P., Brinkman C. R., Johnson G. H., Assali N. S. (1972). Circulatory shock in pregnant sheep. I. Effects of endotoxin on uteroplacental and fetal umbilical circulation.. Am J Obstet Gynecol.

[PDF_01044] Beller F. K., Schmidt E. H., Holzgreve W., Hauss J. (1985). Septicemia during pregnancy: a study in different species of experimental animals.. Am J Obstet Gynecol.

[PDF_01022] Bessey P. Q., Watters J. M., Aoki T. T., Wilmore D. W. (1984). Combined hormonal infusion simulates the metabolic response to injury.. Ann Surg.

[PDF_00990] Beutler B., Cerami A. (1987). Cachectin: more than a tumor necrosis factor.. N Engl J Med.

[PDF_01025] Blair E. (1970). Hypocapnia and gram-negative bacteremic shock.. Am J Surg.

[PDF_00961] Blanco J. D., Gibbs R. S., Castaneda Y. S. (1981). Bacteremia in obstetrics: clinical course.. Obstet Gynecol.

[PDF_01102] Bone R. C., Fisher C. J., Clemmer T. P., Slotman G. J., Metz C. A., Balk R. A. (1987). A controlled clinical trial of high-dose methylprednisolone in the treatment of severe sepsis and septic shock.. N Engl J Med.

[PDF_01067] Boyd J. L., Stanford G. G., Chernow B. (1989). The pharmacotherapy of septic shock.. Crit Care Clin.

[PDF_00967] Bryan C. S., Reynolds K. L., Moore E. E. (1984). Bacteremia in obstetrics and gynecology.. Obstet Gynecol.

[PDF_01032] Carrico C. J., Meakins J. L., Marshall J. C., Fry D., Maier R. V. (1986). Multiple-organ-failure syndrome.. Arch Surg.

[PDF_01138] Cefalo R. C., Lewis P. E., O'Brien W. F., Fletcher J. R., Ramwell P. W. (1980). The role of prostaglandins in endotoxemia: comparisons in response in the nonpregnant, maternal, and fetal models. I. Prostaglandins and the pulmonary effect of experimental endotoxemia.. Am J Obstet Gynecol.

[PDF_01142] Chan C. K., Wells C. K., McFarlane M. J., Feinstein A. R. (1989). More lung cancer but better survival. Implications of secular trends in "necropsy surprise" rates.. Chest.

[PDF_01037] Clark S. L., Cotton D. B., Lee W., Bishop C., Hill T., Southwick J., Pivarnik J., Spillman T., DeVore G. R., Phelan J. (1989). Central hemodynamic assessment of normal term pregnancy.. Am J Obstet Gynecol.

[PDF_01018] Cunnion R. E., Parrillo J. E. (1989). Myocardial dysfunction in sepsis.. Crit Care Clin.

[PDF_01077] Desjars P., Pinaud M., Potel G., Tasseau F., Touze M. D. (1987). A reappraisal of norepinephrine therapy in human septic shock.. Crit Care Med.

[PDF_01090] Duff P., Gibbs R. S. (1983). Pelvic vein thrombophlebitis: diagnostic dilemma and therapeutic challenge.. Obstet Gynecol Surv.

[PDF_00952] Duff P. (1986). Pathophysiology and management of postcesarean endomyometritis.. Obstet Gynecol.

[PDF_01001] Fein A. M., Lippmann M., Holtzman H., Eliraz A., Goldberg S. K. (1983). The risk factors, incidence, and prognosis of ARDS following septicemia.. Chest.

[PDF_01110] Genazzani A. R., Facchinetti F., Parrini D. (1981). beta-lipotrophin and beta-endorphin plasma levels during pregnancy.. Clin Endocrinol (Oxf).

[PDF_00949] Gibbs R. S., Jones P. M., Wilder C. J. (1978). Antibiotic therapy of endometritis following cesarean section. Treatment successes and failures.. Obstet Gynecol.

[PDF_01070] Goldberg L. I. (1974). Dopamine--clinical uses of an endogenous catecholamine.. N Engl J Med.

[PDF_01020] Gonik B. (1986). Septic shock in obstetrics.. Clin Perinatol.

[PDF_01072] Higgins T. L., Chernow B. (1987). Pharmacotherapy of circulatory shock.. Dis Mon.

[PDF_01008] Holaday J. W., Faden A. I. (1978). Naloxone reversal of endotoxin hypotension suggests role of endorphins in shock.. Nature.

[PDF_00988] Jacobs R. F., Tabor D. R. (1989). Immune cellular interactions during sepsis and septic injury.. Crit Care Clin.

[PDF_00992] Kilbourn R. G., Belloni P. (1990). Endothelial cell production of nitrogen oxides in response to interferon gamma in combination with tumor necrosis factor, interleukin-1, or endotoxin.. J Natl Cancer Inst.

[PDF_01034] Knaus W. A., Draper E. A., Wagner D. P., Zimmerman J. E. (1985). Prognosis in acute organ-system failure.. Ann Surg.

[PDF_01132] Lachman E., Pitsoe S. B., Gaffin S. L. (1984). Anti-lipopolysaccharide immunotherapy in management of septic shock of obstetric and gynaecological origin.. Lancet.

[PDF_00964] Ledger W. J., Norman M., Gee C., Lewis W. (1975). Bacteremia on an obstetric-gynecologic service.. Am J Obstet Gynecol.

[PDF_01054] Lee W., Clark S. L., Cotton D. B., Gonik B., Phelan J., Faro S., Giebel R. (1988). Septic shock during pregnancy.. Am J Obstet Gynecol.

[PDF_01121] McCabe W. R., Kreger B. E., Johns M. (1972). Type-specific and cross-reactive antibodies in gram-negative bacteremia.. N Engl J Med.

[PDF_01093] Menashe P. I., Ross S. A., Gottlieb J. E. (1988). Acquired renal insufficiency in critically ill patients.. Crit Care Med.

[PDF_01048] Morishima H. O., Niemann W. H., James L. S. (1978). Effects of endotoxin on the pregnant baboon and fetus.. Am J Obstet Gynecol.

[PDF_00979] Morrison D. C., Ryan J. L. (1987). Endotoxins and disease mechanisms.. Annu Rev Med.

[PDF_01011] Oppenheimer L., Hryniuk W. M., Bishop A. J. (1976). Thrombocytopenia in severe bacterial infections.. J Surg Res.

[PDF_01063] Packman M. I., Rackow E. C. (1983). Optimum left heart filling pressure during fluid resuscitation of patients with hypovolemic and septic shock.. Crit Care Med.

[PDF_01014] Parrillo J. E., Burch C., Shelhamer J. H., Parker M. M., Natanson C., Schuette W. (1985). A circulating myocardial depressant substance in humans with septic shock. Septic shock patients with a reduced ejection fraction have a circulating factor that depresses in vitro myocardial cell performance.. J Clin Invest.

[PDF_01056] Pearlman M., Faro S. (1990). Obstetric septic shock: a pathophysiologic basis for management.. Clin Obstet Gynecol.

[PDF_01087] Pritchard J. A., Whalley P. J. (1971). Abortion complicated by Clostridium perfringens infection.. Am J Obstet Gynecol.

[PDF_00986] Rackow E. C., Astiz M. E. (1991). Pathophysiology and treatment of septic shock.. JAMA.

[PDF_01027] Rawson J. S., Achord J. L. (1985). Shock liver.. South Med J.

[PDF_01084] Reed S. D., Landers D. V., Sweet R. L. (1991). Antibiotic treatment of tuboovarian abscess: comparison of broad-spectrum beta-lactam agents versus clindamycin-containing regimens.. Am J Obstet Gynecol.

[PDF_01107] Rock P., Silverman H., Plump D., Kecala Z., Smith P., Michael J. R., Summer W. (1985). Efficacy and safety of naloxone in septic shock.. Crit Care Med.

[PDF_01080] Schaer G. L., Fink M. P., Parrillo J. E. (1985). Norepinephrine alone versus norepinephrine plus low-dose dopamine: enhanced renal blood flow with combination pressor therapy.. Crit Care Med.

[PDF_01096] Schumer W. (1976). Steroids in the treatment of clinical septic shock.. Ann Surg.

[PDF_00984] Sheagren J. N. (1984). Staphylococcus aureus. The persistent pathogen (first of two parts).. N Engl J Med.

[PDF_01061] Shubin H., Weil M. H., Carlson R. W. (1977). Bacterial shock.. Am Heart J.

[PDF_01074] Sonnenblick E. H., Frishman W. H., LeJemtel T. H. (1979). Dobutamine: a new synthetic cardioactive sympathetic amine.. N Engl J Med.

[PDF_01135] Takayama K., Qureshi N., Ribi E., Cantrell J. L. (1984). Separation and characterization of toxic and nontoxic forms of lipid A.. Rev Infect Dis.

[PDF_01098] Veterans Administration Systemic Sepsis Cooperative Study Group (1987). Effect of high-dose glucocorticoid therapy on mortality in patients with clinical signs of systemic sepsis.. N Engl J Med.

[PDF_00998] Weissmann G., Smolen J. E., Korchak H. M. (1980). Release of inflammatory mediators from stimulated neutrophils.. N Engl J Med.

[PDF_00954] Whalley P. (1967). Bacteriuria of pregnancy.. Am J Obstet Gynecol.

[PDF_01117] Wheeler A. P., Hardie W. D., Bernard G. (1990). Studies of an antiendotoxin antibody in preventing the physiologic changes of endotoxemia in awake sheep.. Am Rev Respir Dis.

[PDF_00981] Wiles J. B., Cerra F. B., Siegel J. H., Border J. R. (1980). The systemic septic response: does the organism matter?. Crit Care Med.

[PDF_01128] Ziegler E. J., Fisher C. J., Sprung C. L., Straube R. C., Sadoff J. C., Foulke G. E., Wortel C. H., Fink M. P., Dellinger R. P., Teng N. N. (1991). Treatment of gram-negative bacteremia and septic shock with HA-1A human monoclonal antibody against endotoxin. A randomized, double-blind, placebo-controlled trial. The HA-1A Sepsis Study Group.. N Engl J Med.

